# Balance Training as an Adjunct to Methylphenidate: A Randomized Controlled Pilot Study of Behavioral Improvement Among Children With ADHD in China

**DOI:** 10.3389/fpsyt.2020.552174

**Published:** 2021-01-08

**Authors:** Lei Feng, Yuanchun Ren, Jia Cheng, Yufeng Wang

**Affiliations:** ^1^Peking University Sixth Hospital, Institute of Mental Health, Beijing, China; ^2^National Clinical Research Center for Mental Disorders and the Key Laboratory of Mental Health, Ministry of Health, Peking University, Beijing, China; ^3^National Clinical Research Center for Mental Disorders and Beijing Key Laboratory of Mental Disorders, Beijing Anding Hospital, Capital Medical University, Beijing, China; ^4^College of Physical Education and Sports, Beijing Normal University, Beijing, China

**Keywords:** ADHD, methylphenidate, balance training, pilot randomized controlled trial, efficacy

## Abstract

**Objective:** This study aimed to compare the therapeutic effects of two different approaches to attention deficit hyperactivity disorder (ADHD): (1) methylphenidate (MPH) treatment combined with balance training, and (2) MPH monotherapy.

**Methods:** The study was based on a randomized, single-blind trial involving 27 ADHD patients. An experimental group received the treatment combining MPH and balance training, while a control group were administered just MPH. After 40 sessions of training at the 6-month mark, patients' improvement as observed in their core symptoms and behavioral problems were compared between the experimental and control group.

**Results:** A total of 27 patients underwent randomization, with 13 assigned to the experimental group and 14 to the control group. After the 6-month trial, the experimental group outperformed the control group in terms of teachers' scores for inattention on the ADHD-RS-IV (19.38 ± 2.96 vs. 23.21 ± 3.91, *t* = −2.854, *P* = 0.009). The experimental group also showed greater improvement on the items involving behavior (3.14 ± 1.46 vs. 5.24 ± 1.04, *t* = 1.463, *P* = 0.026) and hyperactivity (1.92 ± 1.19 vs. 3.86 ± 2.32, *t* = −2.697, *P* = 0.012).

**Conclusion:** In children with ADHD, the experimental group displayed a significant improvement in the symptoms and behavior associated with inattention than did the group whose treatment consisted of only MPH.

## Introduction

Attention deficit hyperactivity disorder (ADHD) is the most prevalent neurodevelopmental disorder among children today. In the United States, approximately 9.4% of children 2–17 years of age have at one time been diagnosed with ADHD, according to parental reports in the 2016 National Survey of Children's Health (1). Among children in China in 2018, the national-pooled prevalence of ADHD of China was 5.6% (2).

ADHD in children is characterized by inattention, hyperactivity, impulsivity, or a combination of these symptoms, which compromise basic everyday functions such as learning to read and making friends (3). The disorder is typically diagnosed in childhood, but affected persons frequently remain symptomatic into adulthood (4, 5). Moreover, ADHD often occurs alongside other disorders and conditions. Observational studies involving 589 patients reported that at least 50% of the ADHD-predominantly inattentive type diagnoses in children accompanied diagnoses of developmental coordination disorder (DCD) (6, 7) and, inversely, 50% of DCD diagnoses in children accompanied ADHD diagnoses (8). These findings indicate that ADHD has a high comorbidity with DCD.

DCD has been shown to affect clinical manifestations of children with ADHD. Previous studies have revealed that children with DCD are more immature, socially isolated, and passive than comparison children, thus restricting their ability to perform daily activities related to self-care and academics (9). A prospective population study with long-term follow up, which compared adolescents who were diagnosed with both ADHD and DCD with a control group after 10 years, found that such adolescents face a higher risk not only of a worsening prognosis but also of psychiatric or personality disorders (10, 11). Therefore, it is vitally important that practitioners distinguish DCD and perform effective interventions as a part of long-term ADHD management.

Stimulants are the most common type of medication used in the treatment of ADHD. Numerous randomized controlled trials have indicated that Methylphenidate offers an effective short-term approach to alleviating core ADHD symptoms, as reported by parents and teachers (12, 13). Furthermore, Flapper et al. (14) and Tucha et al. (15) reported that Methylphenidate can indeed enhance fine motor skills, but that this choice is constrained by the adverse events of this and other stimulants. Rehabilitation strategies, which help children establish the sense of self-control needed for academic and behavioral progress, are emerging as acceptable alternatives (16). In the field of occupational therapy, sensory integration training has been used in children with learning disabilities, ADHD, autism, and behavior problems and found to result in positive outcomes in sensorimotor skills, motor planning, attention regulation and behavioral regulation (17), but there is general agreement that it remains inappropriate for children older than 12 years of age. Therefore, it is necessary to find a solution by which children with ADHD who are in puberty can also succeed.

As children with ADHD usually have poor balance and awkward movements (18, 19), vestibular rehabilitation is a promising and effective alternative (20). Moreover, using a balance platform with biofeedback is sufficient for assessing balance function and can provide quantitative and objective assessments of treatment. This pilot study of children with ADHD was conducted to determine the extent to which treatment combining MPH and balance training may help alleviate the core behavioral symptoms of ADHD in children.

## Materials and Methods

### Trial Design

This study, which combined the treatments of MPH and balance training, was a randomized, single-blind, and parallel-group trial conducted at Peking University Sixth Hospital. The trial was reported in accordance with CONSORT statement. Trial registry number is ChiCTR2000033455 in www.chictr.org.cn.

### Patients

The potential participants for this study were identified by through the psychiatric referrals of outpatients.

To be eligible for this study, patients had to be between 10 and 15 years old and meet three other criteria: (1) have a diagnosis of ADHD in accordance with the Diagnostic and Statistical Manual of Mental Disorders Fourth Edition (DSM-IV); (2) show sensory integrative dysfunction as diagnosed by the Child Sensory Integration Check List; (3) declare that they had been unexposed to any other intervention. Exclusion criteria included a clinical diagnosis of childhood-onset schizophrenia, autism spectrum disorder, intellectual disability, epilepsy, a weight of <30 kg, or an IQ of <80.

### Interventions

After a screening and assessment, all of the participants received oral immediate-release MPH at a starting dose of 5–10 mg once daily with titration in increments of 5–10 mg weekly. The maximum allowed dose was 0.6 mg/kg daily, two or three times daily. The maximum total daily dose was 40 mg. In the event of a serious adverse effect, patients could cease treatment. Then, using of a prospective random-number sequence, the participants who finished their titration and baseline assessment were randomly assigned in a 1:1 ratio, with the experimental group to have balance training as an adjunct to MPH, and with the control group receiving MPH monotherapy. Randomization was performed with permuted blocks of four, and random sequence generation was achieved by using sequentially numbered envelopes. Both the participants and investigators were aware of the study-group assignments. A research psychiatrist who only rated scales and did not participate in training procedure was blind to the assignments.

The balance training machine, which is a pressure platform designed to give visual biofeedback, was Balance Master System and SMART EquiTest System (21), manufactured by Neurocom (Clackamas, Oregon/US) and consisting of three parts: a computer processing system, a training platform (one pressure sensor in each corner), and auxiliary equipment. The procedure for the treatment was based on a one-to-one model that required 30–45 min of training two to four times per week for a total of 40 times. Those patients who failed to cooperate with regular treatment were not included in the study. The goal of treatment was to reach a point where patients had the ability to maintain a balanced posture even when experiencing sensory conflict, the product of inaccurate visual and proprioceptive inputs. While the first 20 training sessions were conducted under the condition of normal visual feedback, the last 20 took place when unreliable feedback weakened the ability to maintain spatial orientation, spatial memory, and spatial posture. For the patients who responded assessed by psychiatrist, the dose was gradually reduced by 5 mg/week, otherwise the dose remained the same as that of the previous week.

### Outcomes

The baseline and follow-up assessments consisted of ADHD-RS-IV, the Rutter Children's Behavior Questionnaire, and the Conners Parent Questionnaire. All of the assessments were performed by trained psychiatrists and psychologists at Peking University Sixth Hospital who remained unaware of the trial-group assignments. The efficacy and safety of the treatments were measured at the initial screening, the baseline assessment, and upon completion of 40 training sessions.

The patients' outcomes were evaluated after 40 training sessions, as suggested by the improvement of both their core symptoms and their behavior. Their core symptoms were evaluated according to ADHD-RS- IV, which included separate forms for parents/caregivers and teachers. Treatment response was defined as a 50% or greater reduction from the baseline. ADHD-RS- IV is a parent-report or teacher-report inventory created by DuPaul et al. (22) consisting of 18 questions regarding a child's behavior over the previous 6 months. The validity of a clinician administration and scoring of the ADHD-RS-IV has been previously demonstrated (23). The Conners Parent Questionnaire (Short Version), consisting of 48 items, was administered to evaluate children's behavior and monitor their response to intervention. The short version provided an evaluation of the six key areas of ADHD: hyperactivity/impulsivity, learning problems, behavioral problems, anxiety, somatic symptom disorder, and an index of hyperactivity. The Conners Teachers Questionnaire was administered to assess children's behavior in school. The Rutter Children Behavior Questionnaire for teachers is often used to determine psychological distress in children, and it consists of 26 simple terms which are divided into “Antisocial Behavior,” “Neurotic Behavior,” and “Mixed Behavior.” The response was defined as a decrease of ≥20% from the baseline total score. Rutter reported that the test showed good re-test and inter-rater reliability (24).

A prespecified outcome was the mean MPH dosage between two groups after finishing 40 training sessions.

### Adverse Events

Specific attention was given to the 14 types of adverse events that were relevant to the MPH drug class. The Modified Barkley Stimulant Side Effect Rating Scale (BSSERS) was administered to children and their parents. The severity of the adverse event was divided into nine grades. We defined a serious adverse event as any event that led to death, was life-threatening, required in-patient hospitalization, prolongation of hospitalization, or resulted in persistent or significant disability, or as any important medical event that may have jeopardized the patient's life or that required intervention. We considered all other adverse events as non-serious (ICH 1996).

### Statistical Analysis

All analyses were performed on an intention-to-treat basis, which included all the patients who had undergone randomization and were confirmed to have received the assigned intervention. The trial data were summarized by the calculation of means and standard deviations for normally distributed variables, medians for non-normally distributed variables, and frequency and percentage for categorical variables. Fisher's exact test was used to compare the proportions of patients who had adverse events in the two groups. We used *t*-tests for continuous variables and a chi-square analysis for categorical variables for comparison of the study groups at baseline. The Mann-Whitney *U*-test was used to compare non-normally distributed variables. All tests for significance were two-sided. The Bonferroni method was used to correct for multitude of comparisons.

## Results

### Participants

**Figure 1:**
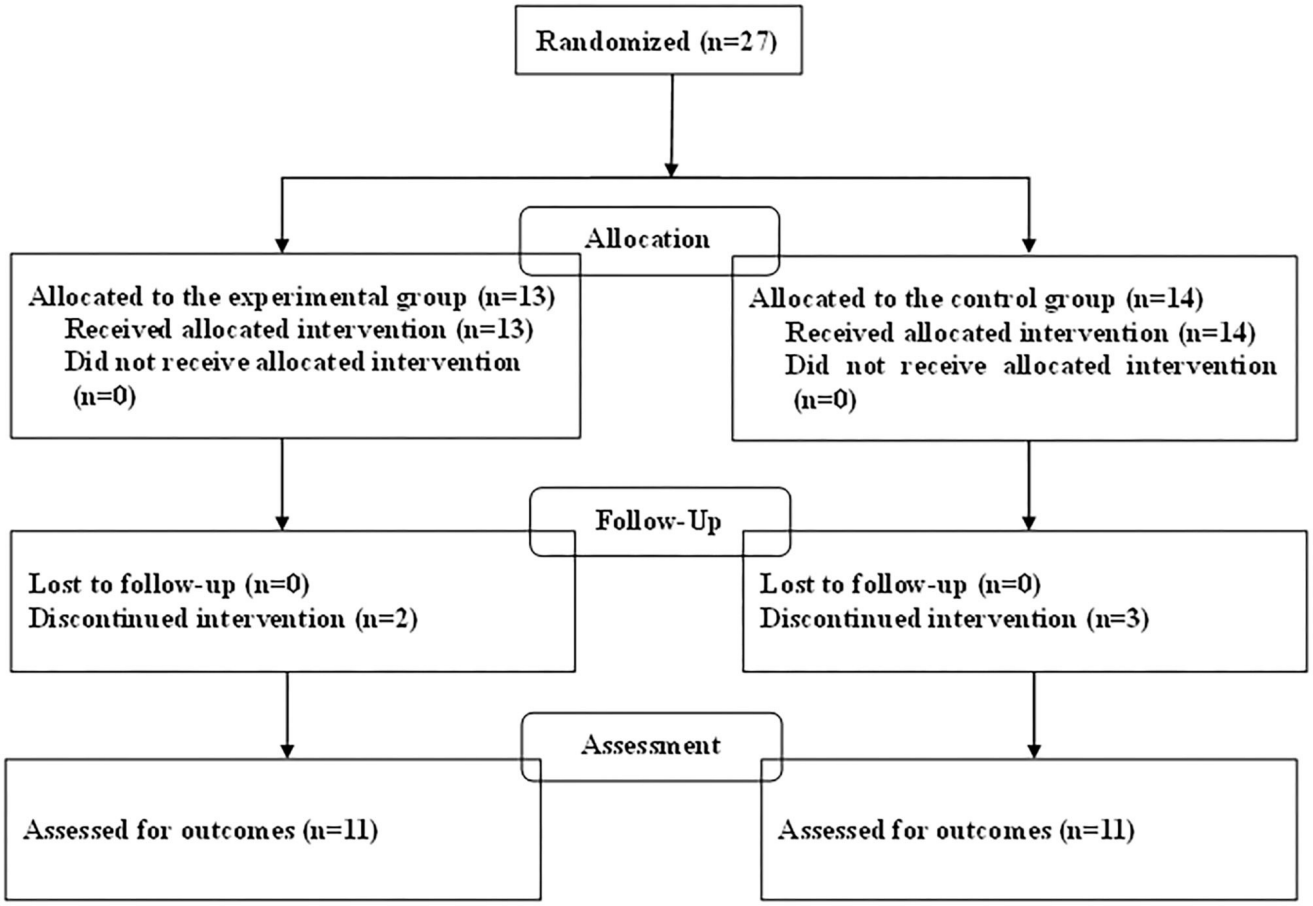
Enrollment flowchart following CONSORT guidelines.

The demographic characteristics of the participants in this study are summarized in Table 1. The baseline characteristics of age, sex, and ADHD subtype were generally shared by the two groups. The total of 27 children with ADHD who were enrolled in the trial consisted of 24 boys (89%) and 3 girls (11%), and of the randomized patients, 13 were allocated to the experimental group and 14 to the control group (Figure 1). In the experimental group receiving combined treatment, all of the children completed both 3 months of training sessions and the second assessment, and 11 completed 40 training sessions and the third assessment. All of the children in the control group completed the second assessment, 1 of them declined medication, and 11 completed the third assessment. Therefore, the study concluded with 11 children in the experimental group and 11 in the control group.

**Table 1:** Demographic and characteristics at baseline by group.

**Characteristic**	**Experimental**	**Control**	***t*/χ^**2**^**	***P*-value**
Age (years)	11.4 ± 1.7	12.3 ± 1.5	−1.36	0.186
Age ranges (years)	10~14.3	10~14.2		
Gender			0.30	1.000
Male	11	13		
Female	2	1		
ADHD subtype			0.31	0.678
ADHD-I	9	11		
ADHD-C	4	3		
**EQUILIBRIUM SCORE OF SENSORY ORGANIZATION TEST**				
SOT condition 1	91.82 ± 1.09	91.03 ± 1.51	0.43	0.672^a^
SOT condition 2	89.07 ± 2.73	89.73 ± 2.65	1.29	0.208^a^
SOT condition 3	89.23 ± 2.99	89.81 ± 4.03	−1.10	0.281^a^
SOT condition 4	82.70	82.92	−1.46	0.145^*^^a^
SOT condition 5	63.80	64.70	−0.29	0.771^*^^a^
SOT condition 6	71.99 ± 6.81	72.34 ± 8.27	−0.80	0.429^a^
SOT composite	82.47 ± 7.36	80.77 ± 4.79	1.56	0.131^a^
Somatosensory ratio	97.89 ± 2.25	96.68 ± 2.89	1.20	0.241^a^
Visual ratio	88.15	92.23	−1.65	0.099^*^^a^
Vestibular ratio	68.75	69.32	−0.44	0.662^*^^a^
Visual dependence	104.27 ± 4.17	107.65 ± 9.24	−1.24	0.230^a^

*ADHD-I, Attention deficit hyperactivity disorder-predominantly inattentive type; ADHD-C, Attention deficit hyperactivity disorder-predominantly combined type; SOT, Sensory Organization Test. Somatosensory Ratio = 100 × SOT2/SOT1; Visual Ratio = 100 × SOT4/SOT1; Vestibular Ratio = 100 × SOT5/SOT1; Visual Dependence =100 × (SOT3+SOT6)/(SOT2 + SOT5)*.

* *Two-sample Mann-Whitney Test*.

a *The adjusted critical level of significance using Bonferroni's correction*.

### Efficacy

This study found it useful to compare and contrast the assessments of parents and teachers. Regarding parents and their ratings on ADHD-RS-IV, there was no significant between-group difference in the response rate after 40 training sessions (4 of 11 patients [36.4%] in the experimental group, 2/11 patients [18.2%] in the control group, *P* > 0.05). Also, the two study groups did not differ significantly with respect to the total score or either of the two subscales (inattention and hyperactivity/impulsivity).

According to the teachers' ratings on ADHD-RS-IV, there was no evidence of a significant difference in the overall response rate between the experimental and control group after 40 training sessions [63.6% (7/11) vs. 27.3% (3/11), *P* = 0.087]. However, the inattention score was significantly lower in the experimental group than it was in the control group (19.38 ± 2.96 vs. 23.21 ± 3.91, *t* = −2.85, *P* = 0.009) at the new critical level of significance after Bonferroni's correction (0.05 ÷ 3 ≈ 0.0167). Moreover, differences of statistical significance were observed in the changes from baseline to endpoint in terms of hyperactivity score (−4.31 ± 4.77 vs. 1.29 ± 8.59, *t* = −2.82, *P* = 0.004), indicating the greater improvement in the experimental group than the control group. The changes from the baseline to the endpoint are summarized in Table 2.

**Table 2:** Summary of outcomes at baseline and endpoint by group.

**Outcomes**	**Experimental**	**Control**	**t/Z**	***P*-value**
**ADHD-RS-IV BY PARENTS**				
**BASELINE**				
Inattention	21.20 ± 3.47	20.17 ± 3.81	0.74	0.469^a^
Hyperactivity/impulsivity	18.21 ± 5.42	19.00 ± 4.72	−0.41	0.687^a^
Total score	36.14 ± 9.45	36.84 ± 7.74	0.69	0.495^a^
**ENDPOINT**				
Inattention	21.20 ± 3.76	21.03 ± 6.00	0.09	0.933^a^
Hyperactivity/impulsivity	16.73 ± 5.43	16.61 ± 5.49	−0.14	0.183^a^
Total score	35.05 ± 5.81	36.45 ± 8.48	−0.85	0.403^a^
**CHANGE FROM BASELINE**				
Inattention	0.00 ± 3.62	0.86 ± 5.26	0.491	0.628^a^
Hyperactivity/impulsivity	−1.48 ± 5.43	−2.39 ± 5.15	−0.447	0.659^a^
Total score	−1.09 ± 8.26	−0.39 ± 8.14	0.222	0.826^a^
**ADHD-RS-IV BY TEACHERS**				
**BASELINE**				
Inattention	23.69 ± 5.75	21.36 ± 4.16	1.22	0.236^a^
Hyperactivity/impulsivity	19.69 ± 5.74	17.57 ± 4.54	1.32	0.179^*^^a^
Total score	36.54 ± 6.39	37.07 ± 8.60	−0.18	0.857^a^
**ENDPOINT**				
Inattention	19.38 ± 2.96	23.21 ± 3.91	−2.85	**0.009^a^^†^**
Hyperactivity/impulsivity	15.38 ± 4.33	18.86 ± 5.56	−1.80	0.084^a^
Total score	28.15 ± 6.15	34.86 ± 10.41	−2.02	0.055^a^
**CHANGE FROM BASELINE**				
Inattention	−4.31 ± 7.47	1.86 ± 6.16	−2.35	0.027^a^
Hyperactivity/impulsivity	−4.31 ± 4.77	1.29 ± 8.59	−2.82	0.004^*^^a^^†^
Total score	−8.38 ± 7.41	−2.21 ± 10.75	−1.72	0.097^a^
**CONNERS FOR PARENTS**				
**BASELINE**				
Behavior	6.45 ± 2.88	8.70 ± 4.85	−1.30	0.208^a^
Learning	5.36 ± 1.63	6.80 ± 2.44	1.71	0.081^*^^a^
Somatic symptom	1.09 ± 1.22	0.70 ± 0.82	−0.60	0.522^*^^a^
Hyperactivity	4.09 ± 2.63	4.60 ± 2.37	0.22	0.802^*^^a^
Anxiety	1.82 ± 1.47	1.60 ± 1.51	−0.41	0.656^*^^a^
Hyperactivity index	10.55 ± 4.44	11.20 ± 4.80	−0.32	0.749^a^
**ENDPOINT**				
Behavior	4.46 ± 3.02	3.14 ± 1.46	1.43	0.171^a^
Learning	4.46 ± 1.76	5.79 ± 2.49	−1.59	0.126^a^
Somatic symptom	0.77 ± 0.73	0.71 ± 0.61	0.11	0.893^*^^a^
Hyperactivity	1.92 ± 1.19	3.86 ± 2.32	−2.76	0.012^a^
Anxiety	2.23 ± 1.09	1.86 ± 1.61	0.57	0.551^*^^a^
Hyperactivity index	5.77 ± 2.28	7.71 ± 2.64	−2.04	0.052^a^
**CHANGE FROM BASELINE**				
Behavior	−1.82 ± 4.07	−5.70 ± 4.74	2.02	0.058^a^
Learning	−0.82 ± 1.40	−1.00 ± 2.00	0.24	0.811^a^
Somatic symptom	−0.36 ± 1.29	0.10 ± 1.10	−0.88	0.388^a^
Hyperactivity	−2.18 ± 2.52	−0.90 ± 1.66	0.98	0.308^*^^a^
Anxiety	0.27 ± 1.85	0.10 ± 2.23	−0.54	0.567^*^^a^
Hyperactivity index	−4.36 ± 3.38	−4.10 ± 4.95	−0.14	0.887^a^
**CONNERS FOR TEACHERS**				
Baseline	11.25 ± 3.69	9.28 ± 4.77	1.33	0.174^*^
Endpoint	6.41 ± 4.27	10.38 ± 4.11	−2.46	**0.021^†^**
Change from baseline	−4.84 ± 5.01	1.10 ± 6.45	−2.66	**0.014^†^**
**RUTTER CHILDREN BEHAVIOR QUESTIONNAIRE BASELINE**				
Total	16.00 ± 5.12	15.71 ± 3.89	0.16	0.871^a^
A behavior	2.08 ± 1.04	2.71 ± 1.07	−1.41	0.151^*^^a^
N behavior	1.62 ± 1.26	1.79 ± 1.12	−0.37	0.713^a^
**ENDPOINT**				
Total	7.31 ± 2.59	10.64 ± 2.24	−3.58	**0.001^a^^†^**
A behavior	1.31 ± 0.75	1.64 ± 1.01	−0.81	0.405^*^^a^
N behavior	0.92 ± 0.76	1.21 ± 1.12	−0.59	0.541^*^^a^
**CHANGE FROM BASELINE**				
Total	−8.69 ± 5.30	−5.07 ± 3.89	−2.03	0.053^a^
A behavior	−0.77 ± 1.24	−1.07 ± 1.00	0.73	0.450^*^^a^
N behavior	−0.69 ± 0.95	−0.57 ± 1.16	0.00	1.000^*^^a^
**METHYLPHENIDATE DOSE**				
Baseline	27.31 ± 5.99	26.79 ± 6.96	0.352	0.706^*^
Endpoint	20.00 ± 5.00	25.00 ± 7.07	−2.05	0.038^*^^†^
Change from baseline	−7.31 ± 4.84	−1.79 ± 4.21	−2.74	0.006^*^^†^

*ADHD-RS-IV, Attention Deficit Hyperactivity Disorder Rating Scale-IV*.

* *Two-sample Mann-Whitney Test*.

a *The adjusted critical level of significance using Bonferroni's correction*.

† *significant*.

*Bold means statistically significant*.

On the Conners Parent Questionnaire (Short Version), the experimental group—more so that the control group—showed a trend toward greater improvement in the items relating to hyperactivity (1.92 ± 1.19 vs. 3.86 ± 2.32, *t* = −2.76, *P* = 0.012) after Bonferroni's correction (0.05 ÷ 6 ≈ 0.0083). In the experimental group, five patients (45.5%) responded to intervention after 40 training sessions, as defined by a 20% reduction in the hyperactivity index, compared with two patients (18.2%) in the control group (*P* > 0.05).

A statistically significant change favoring the experimental group was observed with the Conners Teachers Questionnaire (4.84 ± 5.01 vs. 1.10 ± 6.45, *t* = −2.66, *P* = 0.014, see Table 2).

The total score on the Rutter Children Behavior Questionnaire showed a significant difference between the experimental and control group (7.31 ± 2.59 vs. 10.64 ± 2.24, *t* = −3.58, *P* = 0.001, see Table 2) after 40 training sessions. In the experimental group, five patients (72.7%) responded to intervention after completing the trial, as defined by a 20% reduction in the total Rutter score, compared with two patients (18.2%) in the control group (*P* < 0.05).

### MPH Dose

After 40 training sessions, the mean MPH dose was significantly lower in the experimental group than in the control group (20.00 ± 5.00 vs. 25.00 ± 7.07, *Z* = −2.05, *P* = 0.038, Two-sample Mann-Whitney Test, see Table 2).

### Safety

Not one serious adverse event was reported in this study. In the baseline assessment, adverse events were reported in 84.6% (11/13) of patients in the experimental group and in 85.7% (12/14) in the control group, indicating that there was no significant difference between the two groups in this regard (*P* > 0.05, Fisher's Exact test). Even after 40 training sessions, the difference between the two groups remained small (8/11 vs. 11/11, *P* = 0.062, Fisher's Exact test). The most common non-serious adverse events were sleep problems and a decreased appetite (see Table 3).

**Table 3:** Adverse events by group.

	**Experimental**				**Control**			
**Adverse events**	**N(%)**	**Mild(1–3)**	**Moderate (4–6)**	**Severe(7–9)**	**N(%)**	**Mild(1–3)**	**Moderate (4–6)**	**Severe(7–9)**
**BASELINE**								
Delay of sleep onset	5(38.4%)	3	2	0	2(14.3%)	1	1	0
Nightmares	3(23.1%)	3	0	0	2(14.3%)	2	0	0
Daydreaming	3(23.1%)	2	1	0	5(35.7%)	4	1	0
Talking less to others	4(30.8%)	3	1	0	4(28.6%)	3	1	0
Loss of interest	3(23.1%)	2	1	0	3(21.4%)	1	2	0
Loss of appetite	8(61.5%)	5	1	2	7(50%)	5	1	1
Irritability	8(61.5%)	4	2	2	9(64.3%)	5	2	2
Stomachache	3(23.1%)	2	0	1	3(21.4%)	2	1	0
Headache	3(23.1%)	1	2	0	5(35.7%)	4	1	0
Drowsiness	4(30.8%)	2	2	0	5(35.7%)	5	0	0
Sadness	3(23.1%)	1	2	0	6(42.9%)	4	0	2
Proneness to cry	6(46.2%)	5	1	0	2(14.3%)	0	2	0
Anxiety	4(30.8%)	3	1	0	3(21.4%)	3	0	0
Nail biting	5(38.4%)	3	0	2	4(28.6%)	3	1	0
Euphoria	2(15.4%)	0	2	0	3(21.4%)	3	0	0
Dizziness	3(23.1%)	3	0	0	2(14.3%)	3	0	0
Tics	2(15.1%)	2	0	0	1(7.1%)	1	0	0
**AFTER 6 MONTHS**								
Delay of sleep onset	3(27.3%)	3	0	0	4(36.4%)	2	2	0
Nightmares	2(18.2%)	2	0	0	2(18.2%)	1	0	1
Daydreaming	1(9.1%)	1	0	0	3(27.3%)	2	1	0
Talking less to others	2(18.2%)	1	1	0	3(27.3%)	3	0	0
Loss of interest	2(18.2%)	2	0	0	2(18.2%)	1	1	0
Loss of appetite	6(54.5%)	4	1	1	8(72.7%)	5	1	2
Irritability	6(54.5%)	4	2	0	7(63.6%)	2	3	2
Stomachache	2(18.2%)	1	1	0	1(9.1%)	1	0	0
Headache	3(27.3%)	3	0	0	3(27.3%)	2	1	0
Drowsiness	4(36.4%)	3	1	0	2(18.2%)	1	1	0
Sadness	1(9.1%)	0	1	0	3(27.3%)	2	1	0
Proneness to cry	4(36.4%)	3	1	0	2(18.2%)	2	0	0
Anxiety	3(27.3%)	3	0	0	2(18.2%)	1	1	0
Nail biting	3(27.3%)	3	0	0	4(36.4%)	4	0	0
Euphoria	2(18.2%)	2	0	0	2(18.2%)	2	0	0
Dizziness	2(18.2%)	2	0	0	2(18.2%)	2	0	0
Tics	0(0%)	0	0	0	1(9.1%)	0	1	0

## Discussion

The objective of this pilot study was to explore the efficacy and safety of combining two approaches to the treatment of children with ADHD between 10 and 15 years of age: MPH and balance training, and this pairing was compared with the treatment of using MPH monotherapy.

In this parallel-group trial, the experimental group was associated with statistically significant improvements of their core ADHD symptoms relative to the control group, including the measure of attention, as reported by teachers. These results are consistent with a previous open-label study in which the scores for the symptom of attention deficit showed a significant reduction after 6 months of training (25). Biederman et al. (26) reported that the ADHD symptoms of hyperactivity-impulsive decrease rapidly as biological age increases, while the symptoms of inattention often linger. This may be significant, considering that it is more difficult to treat the symptoms of attention deficit in clinical practice and that symptom improvements are associated with functional improvement (27).

Upon completing the study, the experimental group achieved better results in terms of behavior and hyperactivity, as assessed by parents. The other assessment methods generally reinforced these results, which are consistent with those of previous studies—including those evaluating the improvement of vestibular function, which shows promise in terms of addressing behavioral problems (28, 29). Presumably, balance training may improve children's behavior by enhancing their integration of ontological, visual, and vestibular information, and by its added support to the vestibular system.

The results showed that at the end of 40 training sessions, the dosage of the experimental group decreased while that of the control group remained at a steady level. This difference in dosage was statistically significant. Although the dosage was decreasing, the core symptoms and behavioral problems of children in the experimental group improved more those of the control group. This suggests that balance training may improve the therapeutic effect and facilitate a reduction of the drug's dosage, which verifies our hypothesis. Additionally, dosage reduction has public health significance because a high dosage of stimulants is usually associated with an increased risk of non-serious adverse events (30).

This trial has several strengths. First, the randomized controlled design was adopted to prevent the skewing of participants' selection. Second, quality control during the trial was strict. All of the physicians involved in drug titration were psychiatrists with extensive experience in clinical work. Similarly, all of the researchers had been trained to the same standards before the start of the trial and then continuously evaluated for consistency. Third, we assessed the comprehensive effects of the intervention.

This trial also had several limitations. First, the sample size was small. And small sample size may increase the likelihood of a Type II error skewing the results, which decreases the statistical power of the study. Second, the design of randomized control trial could have lacked external validity, thus rendering invalid any generalizations of its findings to populations outside the study group. Third, our study did not have the time frame required to assess the duration of treatment effect or to identify rare adverse effects. Forth, personal interaction in the experimental group may have positive effect on the children's behavior. Fifth, our study lacked a control group of healthy children in balance training who did not have ADHD.

In conclusion, in this randomized trial involving patients with ADHD, adding balance training to MPH significantly reduced symptoms of inattention and ameliorated behavioral problems more than did the use of MPH monotherapy. In terms of diminishing adverse events, our combined treatment did not yield significantly greater benefits but may have provided this group with just enough of an advantage such that their MPH dosage could be decreased. These data suggest that further studies are needed to assess the long-term outcomes for the two groups with a reasonably large sample size.

## Data Availability Statement

The data analyzed in this study is subject to the following licenses/restrictions: These data are provided for the purpose of statistical reporting and analysis only. Requests to access these datasets should be directed to Yufeng Wang, wangyf@bjmu.edu.cn.

## Ethics Statement

The studies involving human participants were reviewed and approved by Institutional review boards at Peking University Health Science Center. Written informed consent to participate in this study was provided by the participants' legal guardian/next of kin.

## Author Contributions

LF: study design, study management, recruitment, data analysis, data interpretation, drafting manuscript and revisions, and final approval of the article. YR and JC: patient enrollment, collection and assembly of data, data interpretation, and final approval of the article. YW: obtain funding, study design, article review and revisions, and final approval of the article. All authors contributed to the article and approved the submitted version.

## Conflict of Interest

The authors declare that the research was conducted in the absence of any commercial or financial relationships that could be construed as a potential conflict of interest. The reviewer HD declared a shared affiliation with several of the authors, LF, JC, YW, to the handling editor at time of review.

## References

[1] Danielson ML Bitsko RH Ghandour RM Holbrook JR Kogan MD Blumberg SJ. Prevalence of parent-reported ADHD diagnosis and associated treatment among U.S. children and adolescents, 2016. J Clin Child Adolesc Psychol. (2018) 47:199–212. 10.1080/15374416.2017.1417860 29363986 PMC5834391

[2] Li SM Feng W Fang F Dong XH Zhang ZJ Yang QQ. Prevalence of attention deficit and hyperactivity disorder in children in China: a systematic review and Meta-analysis. Zhonghua Liu Xing Bing Xue Za Zhi. (2018) 39:993–8. 10.3760/cma.j.issn.0254-6450.2018.07.024 30060318

[3] American Psychiatric Association DSM-V (Diagnostic and Statistical Manual of Mental Disorders). 5th ed American Journal of Psychiatry (2013) 61.

[4] Kooij SJJ Bejerot S Blackwell A Caci H Casas-Brugué M Carpentier PJ. European consensus statement on diagnosis and treatment of adult ADHD: The European Network Adult ADHD. BMC Psychiatry. (2010) 10:67. 10.1186/1471-244X-10-67 20815868 PMC2942810

[5] Biederman J Petty CR Woodworth KY Lomedico A Hyder LL Faraone S V. Adult outcome of attention-deficit/hyperactivity disorder: A controlled 16-year follow-up study. J Clin Psychiatry. (2012) 73:941–50. 10.4088/JCP.11m07529 22901345

[6] Landgren M Pettersson R Kjellman B Gillberg C. ADHD, DAMP and other neurodevelopmental/psychiatric disorders in 6-year-old children: epidemiology and co-morbidity. Dev Med Child Neurol. (2008) 38:891–906. 10.1111/j.1469-8749.1996.tb15046.x 8870611

[7] Watemberg N Waiserberg N Zuk L Lerman-Sagie T. Developmental coordination disorder in children with attention-deficit-hyperactivity disorder and physical therapy intervention. Dev Med Child Neurol. (2007) 49:920–5. 10.1111/j.1469-8749.2007.00920.x 18039239

[8] Kadesjö B Gillberg C. Developmental coordination disorder in Swedish 7-year-old children. J Am Acad Child Adolesc Psychiatry. (1999) 38:820–8. 10.1097/00004583-199907000-00011 10405499

[9] Masi L ADHD and comorbid disorders in childhood psychiatric problems, medical problems, learning disorders and developmental coordination disorder. Clin Psychiatry. (2015) 1:5 10.21767/2471-9854.100005

[10] Hellgren L Gillberg C Gillberg IC Enerskog I. Children with deficits in attention, motor control and perception (damp) almost grown up: General health at 16 years. Dev Med Child Neurol [Internet]. (2008) 35:881–92. 10.1111/j.1469-8749.1993.tb11565.x 8405717

[11] Hellgren L Carina Gillberg I Bågenholm A Gillberg C. Children with deficits in attention, motor control and perception (DAMP) almost grown up: psychiatric and personality disorders at age 16 years. J Child Psychol Psychiatry [Internet]. (1994) 35:1255–71. 10.1111/j.1469-7610.1994.tb01233.x 7806609

[12] Parker J Wales G Chalhoub N Harpin V The long-term outcomes of interventions for the management of attention-deficit hyperactivity disorder in children and adolescents: a systematic review of randomized controlled trials. Psychol Res Behav Manag. (2013) 17:87–99. 10.2147/PRBM.S49114 PMC3785407 24082796

[13] Storebø OJ Ramstad E Krogh HB Nilausen TD Skoog M Holmskov M. Methylphenidate for children and adolescents with attention deficit hyperactivity disorder (ADHD). Cochrane Database Syst Rev. (2015) 25:CD009885. 10.1002/14651858.CD009885.pub2 26599576 PMC8763351

[14] Flapper BCT Houwen S Schoemaker MM. Fine motor skills and effects of methylphenidate in children with attention-deficit—hyperactivity disorder and developmental coordination disorder. Dev Med Child Neurol. (2006) 48:165–9. 10.1017/S0012162206000375 16483390

[15] Tucha O Lange KW. Handwriting and attention in children and adults with attention deficit hyperactivity disorder. Motor Control. (2004) 8:461–71. 10.1123/mcj.8.4.461 15585901

[16] DuPaul GJ White GP ADHD: behavioral, educational, and medication interventions. Educ Dig. (2006) 71:57–60.

[17] May-Benson TA Koomar JA. Systematic review of the research evidence examining the effectiveness of interventions using a sensory integrative approach for children. Am J Occup Therapy. (2010) 64:403–14. 10.5014/ajot.2010.09071 20608272

[18] Raberger T Wimmer H. On the automaticity/cerebellar deficit hypothesis of dyslexia: Balancing and continuous rapid naming in dyslexic and ADHD children. Neuropsychologia. (2003) 41:1493–7. 10.1016/S0028-3932(03)00078-2 12849767

[19] Tseng MH Henderson A Chow SMK Yao G. Relationship between motor proficiency, attention, impulse, and activity in children with ADHD. Dev Med Child Neurol. (2004) 46:381–8. 10.1017/S0012162204000623 15174529

[20] Aminjan AS Hosseini SA Haghgoo HA Rostami R Vestibular stimulation and auditory perception in children with attention deficit hyperactivity disorder. Iran Rehabil J. (2014) 12:39–42. 10.13140/RG.2.1.4473.5842

[21] Chaudhry H Bukiet B Ji Z Findley T. Measurement of balance in computer posturography: comparison of methods—A brief review. J Bodyw Mov Ther. (2011) 15:82–91. 10.1016/j.jbmt.2008.03.003 21147423

[22] DuPaul GJ Power TJ Anastopoulos AD Reid R ADHD Rating Scale-IV: Checklists, Norms, and Clinical Interpretation. New York, NY: Guilford Press (1998).

[23] Faries DE Yalcin I Harder D Heiligenstein JH. Validation of the ADHD rating scale as a clinician administered and scored instrument. J Atten Disord. (2001) 5:107–15. 10.1177/10870547010050020416395872

[24] Rutter M. A children's behaviour questionnaire for completion by teachers: preliminary findings. J Child Psychol Psychiatry [Internet]. (1967) 8:1–11. 10.1111/j.1469-7610.1967.tb02175.x 6033260

[25] Cheng J Wang Y Observation of behavior problem and posture stability before and after visual feedback balance training in children with attention deficit hyperactivity disorder. Chinese J Clin Rehabil. (2005) 9:58–61. 10.3321/j.issn:1673-8225.2005.48.023

[26] Biederman J Mick E Faraone SV. Age-dependent decline of symptoms of attention deficit hyperactivity disorder: Impact of remission definition and symptom type. Am J Psychiatry. (2000) 157:816–8. 10.1176/appi.ajp.157.5.816 10784477

[27] Steele M Jensen PS Quinn DMP. Remission versus response as the goal of therapy in ADHD: a new standard for the field? Clin Ther. (2006) 28:1892–908. 10.1016/j.clinthera.2006.11.006 17213010

[28] Bhatara V Clark DL Arnold LE Gunsett R Smeltzer DJ. Hyperkinesis treated by vestibular stimulation: an exploratory study. Biol Psychiatry. (1981) 16:269–79. 7225490

[29] Arnold LE Clark DL Sachs LA Jakim S Smithies C. Vestibular and visual rotational stimulation as treatment for attention deficit and hyperactivity. Am J Occup Ther Off Publ Am Occup Ther Assoc. (1985) 39:84–91. 10.5014/ajot.39.2.84 3872075

[30] Ahmann PA Waltonen SJ Olson KA Theye FW Van Erem AJ LaPlant RJ. Placebo-controlled evaluation of Ritalin side effects. Pediatrics. (1993) 91:1101–6. 8502509

